# Intake of Potatoes Is Associated with Higher Diet Quality, and Improved Nutrient Intake and Adequacy among US Adolescents: NHANES 2001–2018 Analysis

**DOI:** 10.3390/nu13082614

**Published:** 2021-07-29

**Authors:** Sanjiv Agarwal, Victor L. Fulgoni

**Affiliations:** 1 NutriScience LLC, East Norriton, PA 19403, USA; 2 Nutrition Impact, LLC, Battle Creek, MI 49014, USA; VIC3RD@aol.com

**Keywords:** Healthy Eating Index, HEI, minerals, vitamins

## Abstract

Potatoes are nutrient rich white vegetables, however, research on their impact on public health is limited. The objective of this study was to provide updated evaluation of the cross-sectional association between potato consumption and diet quality, nutrient intake and adequacy. Twenty-four hour diet recall data from adolescents (*n* = 16,633; age 9–18 years) were used to assess intakes. Usual intakes of nutrients were determined using the National Cancer Institute method and diet quality was calculated using the Healthy Eating Index-2015 (HEI-2015) after adjusting for demographic factors. Consumers of potatoes (baked or boiled potatoes, mashed potatoes and potato mixtures, fried potatoes, and potato chips) had higher (*p* < 0.05) HEI-2015 total score and subcomponent scores for total vegetables, total protein foods, and refined grain than non-consumers. Consumers also had higher (*p* < 0.05) intake of energy, dietary fiber, protein, copper, magnesium, phosphorus, potassium, selenium, sodium, zinc, niacin, vitamin B_6_, vitamin C, vitamin K and total choline; and higher (*p* < 0.05) adequacy for protein, copper, magnesium, phosphorus, potassium, zinc, thiamine, niacin, vitamin B_6_, vitamin C, and vitamin K than non-consumers. In conclusion, adolescent potato consumption was associated with higher diet quality, nutrient intake, and adequacy and therefore encouraging their consumption may be an effective strategy for improving nutritional status.

## 1. Introduction

Potatoes are the most widely consumed non-cereal staple food consumed worldwide [1]. They are nutrient-rich, typically white, vegetables providing significant amounts of key essential nutrients, including dietary fiber, potassium, magnesium, vitamin C, vitamin B6, and phytonutrients [2,3,4] and are a more affordable source of nutrients with more favorable overall nutrient-to-price ratio compared to many other vegetables [5]. While potatoes are regarded as vegetables in United States (US) dietary recommendations [6], they are not considered as vegetables by World Health Organization [7] and are grouped as cereals in the United Kingdom National Food guide [8]. In the US, potato consumption is over 100 pounds per year per capita, accounting for ~30% of total vegetable intake [9]. Their intake was estimated to be 0.35 cup equivalents/day/person in an analysis of National Health and Nutrition Examination Survey (NHANES) 2009–2010 [10].

Potatoes are classified as starchy vegetables because carbohydrates and especially starch (amylose, amylopectin, and resistant starch) are their predominant macronutrient. While potatoes are rich in carbohydrates (starch), because of their high amount of water they have a low energy density compared to other carbohydrate sources [11]. Cooking, cooling, and re-heating increases resistant starch levels in potatoes. Emerging research suggests that resistant starch may enhance satiety, may affect body composition, blood lipid and blood glucose levels; and stimulate growth and increase number of pro-bacteria in the colon [12,13].

Current scientific research on potato consumption and its impact on public health is controversial. In prospective studies, consumption of potatoes was associated with higher risk of type 2 diabetes and hypertension [14,15]. However, a systematic review published in 2016 did not find any conclusive evidence suggesting an association between potato intake and the risk of developing obesity and type 2 diabetes [16]. A recently published NHANES 1999–2010 data analysis reported positive association of higher potato intake with cardiometabolic risk factors but did not find any significant effects on long-term mortality rates [17]. However, only very limited data are available and a research gap exists in children and adolescents.

White potatoes represented about a third of all vegetable servings consumed by US children and adolescents in an analysis of NHANES 2003–2008 [18]. In another analysis of NHANES 2003–2006, white potatoes including French fries contributed to intake of energy and several shortfall nutrients in the diets of children and adolescents [19]. A dietary modeling analysis of NHANES 2005–2012 indicated that removing potatoes from children’s diet might compromise potassium intake [20]. Federal nutrition policies related to the role of white potatoes in the diet of adolescents have been controversial and continuously evolving. In 2009, the Institute of Medicine (IOM) and United States Department of Agriculture (USDA) recommended to limit/excluded potatoes and other starchy vegetables from school meal plan [21,22]; however, in 2012, the proposal was struck down by the US Senate prohibiting USDA from setting any maximum limits on the serving of vegetables in school meal programs and removing the restriction on starchy vegetables (including French fries) in school lunches [22]. Starchy vegetables, including potatoes, were also initially restricted to one cup per week in the Special Supplemental Nutrition Program for Women, Infants, and Children (WIC) and the Health Hunger Free Kids Act [23] and in 2009 the WIC program offered a monthly fruit and vegetable cash value voucher that excluded white potatoes [24]; however, an IOM report in 2015 [25] indicated that white potatoes are particularly high in potassium and their increased consumption may help reduce shortfalls of potassium in the diets of both children and women, and recommended that they should be included as eligible vegetables under WIC program. In a recent analysis of longitudinal data from the National Heart, Lung, and Blood Institute’s Growth and Health Study, intake of potassium, magnesium, vitamin B6, and vitamin C increased with increasing intake of potatoes at the baseline in adolescent girls [26].

Given this background, we hypothesize that potatoes as nutrient rich vegetables would be associated with improved diet quality and nutrient intake in all adolescents. Therefore, the purpose of this study was to provide an updated evaluation of the association of potato consumption among adolescents on diet quality, nutrient intake, and nutrient adequacy using NHANES 2001–2018 data. To separate the effects of less processed potatoes from more processed potatoes, we used three different definitions to define potato consumers—(1) those consuming potatoes as baked, boiled, and mashed and mixtures; (2) those consuming potatoes as baked, boiled, mashed and mixtures, and fried; and (3) those consuming potatoes as baked, boiled, mashed and mixtures, fried, and chips.

## 2. Materials and Methods 

NHANES, a continuous large survey of a nationally representative sample of the non-institutionalized US population conducted by the National Center for Health Statistics, were used to assess potato intake [27]. 24 h dietary recall data of 16,633 adolescents 9–18 years participating in nine cycles of NHANES 2001–2018 were combined for the analyses to increase the sample size. Data from pregnant or lactating females and those with unreliable or incomplete data determined by the USDA were excluded. NHANES data are collected using a complex stratified multistage cluster sampling probability design. A detailed description of the subject recruitment, survey design, and data collection procedures is available online [27], and all data obtained for this study are publicly available at http://www.cdc.gov/nchs/nhanes/, (accessed on 11 December 2020). All participants or proxies provided written informed consent and the Research Ethics Review Board at the National Center for Health Statistics approved the survey protocol. NHANES has stringent consent protocols and procedures to ensure confidentiality and protection from identification. This study was a secondary data analysis which lacked personal identifiers, therefore, did not require Institutional Review Board review.

Dietary intake of energy, nutrients, and food components was determined using 24-h dietary recalls, which included an in-person interview in the Mobile Examination Center (day 1 recall), followed by a telephone interview conducted 3–10 days after (day 2 recall). Parents or guardians assisted 9–11 years old adolescents, while 12–18 years old adolescents provided their own recalls. Participants were dichotomized into consumers and non-consumers of potatoes; and consumers were further classified into intake quartiles based on individual usual intakes. Consumers were defined as those individuals consuming any amount of potatoes on either of the two days of dietary recall. Potato intakes were assessed using a total of four What We Eat in America (WWEIA) food categories (numbers indicated assigned by USDA for categorization purposes): baked or boiled potatoes (#6802), mashed potatoes and potato mixtures (#6806), fried potatoes (#6804), and potato chips (#5002). We used three definitions for defining potato intake:

Case 1: included WWEIA categories 6802 (baked or boiled), 6806 (mashed and mixtures)

Case 2: included WWEIA categories 6802, 6806, 6804 (fried)

Case 3: included WWEIA categories 6802, 6806, 6804, 5002 (chips)

Demographic information and physical activity levels were determined from the NHANES interview [27].

Diet quality scores were determined using the USDA Healthy Eating Index–2015 (HEI-2015) [28], which has 13 subcomponents, each reflecting an aspect of the 2015–2020 Dietary Guidelines for Americans [29] recommendations. Dietary intake was expressed per 1000 kcal for all subcomponents, except for fatty acid ratios, which were expressed as ratio of unsaturated to saturated fatty acids, and for saturated fat and added sugars, both expressed as % energy. Subcomponents for total vegetables; greens and beans; total fruit, whole fruit; total protein; and seafoods and plant proteins were scored proportionally from 0 to 5 points and all other subcomponents (i.e., whole grains; dairy; fatty acids; sodium; refined grains; saturated fat; and added sugars) were scored proportionally from 0 to 10 points. Four subcomponents, sodium, refined grains, saturated fat, and added sugars were reverse scored, so that lower intake leads to a higher score, and thus a greater contribution to overall diet quality. The maximum possible score was 100 [28]. The intake on the first dietary recall were used to determine HEI-2015 total and subcomponent scores.

Energy and nutrient intake were determined using the NHANES cycle specific USDA Food & Nutrient Database for Dietary Studies [30,31]. Food components to assess HEI were determined using the NHANES cycle specific USDA MyPyramid Servings Database Food or Food Patterns Equivalents Database [32,33].

Analyses were performed using SAS 9.4 and data adjusted for the complex sampling (clustered sample) design of NHANES, using appropriate survey weights, strata, and primary sampling units. Least-square means (and the standard errors of the least-square means) for diet quality (HEI-2015 score), and for energy and nutrient intake were generated using regression analyses adjusted for key covariates (age, gender, and ethnicity). Usual intakes of energy, nutrients, and food groups were determined using the National Cancer Institute (NCI) method and NCI macros were used to estimate distribution of usual intake [34]. Nutrient adequacy was estimated by assessing the percentage of the population below the Estimated Average Requirement (EAR) or above Adequate Intake (AI) of nutrients using the cut-point method (except for iron, where the probability method was used, which is the recommended approach when assessing iron intake given asymmetrical requirement distribution of iron in menstruating women [35]). Significant differences between consumers and non-consumers were assessed via t-test and regression analyses were used to assess the linear relationship of potato intake quartiles with diet quality and nutrient intake.

## 3. Results

**Figure 1:**
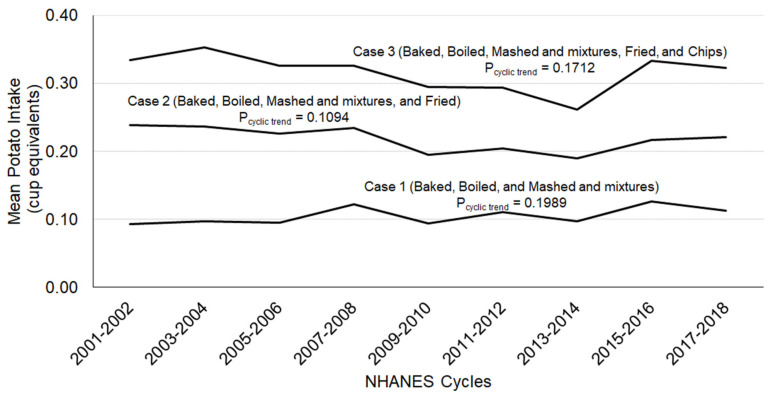
Covariate (age, gender, and ethnicity) adjusted mean intake of potatoes among adolescents (9–18 years of age, *n* = 16,633) by NHANES study periods; gender combined data.

Mean per capita intake of potatoes for Case 1 (baked, boiled, and mashed and mixtures), Case 2 (baked, boiled, mashed and mixtures, and fried), and Case 3 (baked, boiled, mashed and mixtures, fried, and chips) were 0.11 ± 0.01, 0.22 ± 0.01, and 0.32 ± 0.01 cup eq, respectively, for NHANES 2001–2018. Mean per capita intake of potatoes (for all three definitions) was stable and did not change significantly (*p* > 0.05) over the last 18 years (9 NHANES cycles) among US adolescents (Figure 1).

Demographics: Approximately 15.2% of adolescents were Case 1 potato (baked, boiled, mashed and mixtures) consumers. A lower (p < 0.05) proportion of potato consumers were male (−10.3%), Mexican American (−18.3%), other Hispanic (−28.4%), non-Hispanic Black (−16.4%), other ethnicity (−23.1%), and overweight (−13.0%), while a greater proportion of Case 1 potato consumers were non-Hispanic White (+16.1%) as compared to non-consumers (Table 1). All other demographic characteristics evaluated were similar (*p* > 0.05) among consumers and non-consumers potatoes in Case 1 (Table 1).

Approximately 43.2% and 56.5% of adolescents were potato consumers by potato intake definition Case 2 (baked, boiled, mashed and mixtures, and fried) and Case 3 (baked, boiled, mashed and mixtures, fried, chips), respectively. For these potato definitions a lower (*p* < 0.05) proportion of adolescent consumers were male (−7.85% and −6.13%, respectively), Mexican American (−17.4% and −20.0%, respectively), other Hispanic (−18.3% and −20.1%, respectively), other ethnicity (−15.0% and −13.6% respectively), and below HS education (−0.71% and −0.71%, respectively), and a higher (*p* < 0.05) proportion were (*p* < 0.05) non-Hispanic Black (+21.5% and +36.4%, respectively) and had a HS education (+61.3% and +54.8%, respectively). All other demographic characteristics evaluated were similar (*p* > 0.05) among consumers and non-consumers of potatoes in Case 2 and 3 (Table 1).

Diet Quality: Adolescent consumers of potatoes in Case 1 (baked, boiled, mashed and mixtures) as compared to non-consumers had higher HEI-2015 total score (+4.70%, *p* < 0.01), and the score gradually increased with increasing intake quartile (*β* = 0.77 ± 0.15, P_quartile trend_ < 0.01). HEI-2015 subcomponent scores among potato consumers compared to non-consumers were also higher (*p* < 0.05) for total vegetables (+49.5%), total protein foods (+9.22%), refined grain (+24.2%), and added sugar (+5.76%); and gradually increased with increasing intake quartiles (*β* = 0.39 ± 0.02, *β* = 0.09 ± 0.02, *β* = 0.42 ± 0.05, *β* = 0.13 ± 0.04, respectively; P_quartile trend_ < 0.01 for all). However, subcomponent scores among Case 1 potato consumers compared to non-consumers were lower for dairy and sodium (−5.61%, −10.1%, respectively; *p* < 0.01 for both) and gradually decreased with increasing intake quartiles (*β* = −0.15 ± 0.03, *β* = −0.17 ± 0.03, respectively; P_quartile trend_ < 0.01 for both) (Table 2).

Adolescent consumers of potatoes in Case 2 (baked, boiled, mashed, and mixtures, fried) as compared to non-consumers also had a higher (2.01%, *p* < 0.01) HEI-2015 total score, and higher (*p* < 0.01) subcomponent scores for total vegetables (36.8%), total protein foods (7.39%), fatty acid ratio (16.5%), and refined grain (27.5%). However, subcomponent scores among Case 2 potato consumers compared to non-consumers were lower (*p* < 0.01) for greens and beans (−20.0%), total fruit (−11.5%), whole fruit (−13.4%), whole grain (−18.5%), dairy (−8.64%), and seafood and plant protein (−11.9%) (Table 3).

Adolescent consumers of potatoes in Case 3 (baked, boiled, mashed and mixtures, fried, chips) as compared to non-consumers had a higher (1.57%, *p* < 0.05) HEI-2015 total score, and higher (*p* < 0.01) subcomponent scores for total vegetables (41.2%), total protein foods (5.38%), fatty acid ratio (18.4%) and refined grain (26.9%); and lower (*p* < 0.01) subcomponent score for greens and beans (−26.5%), total fruit (−8.72%), whole fruit (−11.7%), whole grain (−22.2%), dairy (−8.97%), seafood and plant protein (−11.1%), added sugar (−4.81%) (Table 3).

Nutrient Intake: Adolescent consumers of potatoes in Case 1 (baked, boiled, mashed and mixtures) as compared to non-consumers had higher (*p* < 0.05) intake of energy (+6.26%), carbohydrate (+5.43%), dietary fiber (+8.51%), protein (+12.0%), copper (+12.4%), magnesium (+10.3%), phosphorus (+6.62%), potassium (+18.7%), selenium (+6.86%), sodium (+8.98%), zinc (+9.09%), vitamin A (+9.59%), thiamine (+4.24%), niacin (+11.7%), vitamin B_6_ (+20.8%), vitamin C (+23.1%), vitamin K (+22.2%), and total choline (+14.9%). Intakes of all other nutrients evaluated were similar (*p* > 0.05) among consumers and non-consumers (Table 4).

Increasing intake quartiles also gradually increased (P_quartile trend_ < 0.05) intake of energy (*β* = 51.9 ± 10.2 kcal), carbohydrate (*β* = 5.86 ± 1.47 mg), dietary fiber (*β* = 0.55 ± 0.09 g), protein (*β* = 3.36 ± 0.50 g), copper (*β* = 0.06 ± 0.01 mg), magnesium (*β* = 10.6 ± 1.6 mg), phosphorus (*β* = 33.8 ± 7.2 mg), potassium (*β* = 174 ± 12 mg), selenium (*β* = 2.75 ± 0.75 μg), sodium (*β* = 116 ± 18 mg), zinc (*β* = 0.43 ± 0.08 mg), vitamin A (*β* = 23.2 ± 7.2), thiamine (*β* = 0.03 ± 0.01 mg), riboflavin (*β* = 0.03 ± 0.01 mg), niacin (*β* = 1.08 ± 0.18 mg), vitamin B_6_ (*β* = 0.15 ± 0.01 mg), vitamin C (*β* = 7.36 ± 1.73 mg), vitamin E (*β* = 0.16 ± 0.08 mg), vitamin K (*β* = 5.62 ± 1.46 μg), and total choline (*β* = 15.1 ± 2.2 mg) (Table 4).

Adolescent consumers of potatoes in Case 2 (baked, boiled, mashed and mixtures, fried) as compared to non-consumers had higher (*p* < 0.05) intake of energy (+11.5%), carbohydrate (+9.33%), dietary fiber (+6.47%), protein (+8.94%), copper (+8.74%), magnesium (+5.81%), phosphorus (+6.41%), potassium (+16.8%), selenium (+2.94%), sodium (+10.6%), zinc (+6.42%), niacin (+11.2%), vitamin B_6_ (+15.0%), vitamin C (+8.91%), vitamin E (+5.72%), vitamin K (+9.26%), and total choline (+11.3%); and lower (*p* < 0.05) intakes of vitamin A (−4.62%) and folate (−5.44%). Intakes of all other nutrients evaluated were similar (*p* > 0.05) among consumers and non-consumers of Case 2 potatoes (Table 5).

Adolescent consumers of potatoes in Case 3 (baked, boiled, mashed and mixtures, fried, chips) as compared to non-consumers had higher (*p* < 0.01) intake of energy (+11.8%), carbohydrate (+9.85%), dietary fiber (+5.04%), protein (+7.02%), copper (+8.82%), magnesium (+5.86%), phosphorus (+5.42%), potassium (+18.5%), sodium (+10.1%), zinc (+5.50%), niacin (+9.87%), vitamin B_6_ (+14.0%), vitamin C (+11.9%), vitamin E (+15.2%), and total choline (+7.36%); and lower (*p* < 0.01) intakes of vitamin A (−5.71%) and folate (−4.90%). Intakes of all other nutrients evaluated were similar (*p* > 0.05) among consumers and non-consumers of Case 3 potatoes (Table 5).

Nutrient Adequacy: Compared to non-consumers, a lower (*p* < 0.05) proportion of adolescent consumers of potatoes in Case 1 (baked, boiled, mashed and mixtures) were below the EAR for carbohydrate, protein, copper, iron, magnesium, phosphorus, selenium, zinc, vitamin A, thiamine, riboflavin, niacin, vitamin B_6_ and vitamin C, and a higher (*p* < 0.05) proportion were above AI for potassium, sodium, and vitamin K (Table 6).

Similarly, compared to non-consumers, a lower (*p* < 0.05) proportion of adolescent consumers of potatoes in Case 2 (baked, boiled, mashed and mixtures, fried) were below the EAR for carbohydrate, protein, copper, magnesium, phosphorus, zinc, thiamine, riboflavin, niacin, vitamin B_6_, vitamin B_12_ and vitamin C; and a higher (*p* < 0.05) proportion were above AI for potassium, sodium, and vitamin K. However, a higher (*p* < 0.05) proportion of consumers compared to non-consumers were below the EAR for calcium, vitamin A, and vitamin D; and a lower (*p* < 0.05) proportion were above AI for dietary fiber (Table 6).

Compared to non-consumers, a lower (*p* < 0.05) proportion of adolescent consumers of potatoes in Case 3 (baked, boiled, mashed and mixtures, fried, chips) also were below the EAR for carbohydrate, protein, copper, iron, magnesium, phosphorus, zinc, thiamine, niacin, vitamin B_6_, vitamin B_12_ and vitamin C, and a higher (*p* < 0.05) proportion were above AI for potassium, sodium, and vitamin K. However, a higher (*p* < 0.05) proportion of consumers compared to non-consumers were below the EAR for calcium, vitamin A, and vitamin D (Table 6).

## 4. Discussion

In the current cross-sectional analysis of data from nine cycles of NHANES (NHANES 2001–2018) using a nationally representative sample of over 16,000 US adolescents, potato consumption was associated with better diet quality, higher intake, and adequacy of several nutrients, including shortfall nutrients.

Potatoes are part of the starchy vegetables subgroup and are present in many different forms representing various cooking/processing methods in the diet. Potato chips, boiled potatoes, fries (French fries and home fries), and baked potatoes represent 28.7%, 23.5%, 22.3%, 10.8%, respectively, of starchy vegetables intake among children age 4–18 years [36]. Limited data suggest that different forms of potatoes may have different nutritional and/or health attributes/outcomes [37,38,39,40]. For example, baked potatoes have a low glycemic index due to their high amount of resistant starch–a dietary fiber [41,42]. Potato chips are often considered as junk food, and fried potatoes may contain acrylamide, which is a potential carcinogen [43]. In the present analysis, we used three different definitions to define potato consumers—Case 1: those consuming baked, boiled, mashed, and mixtures; Case 2: those consuming baked, boiled, mashed and mixtures, fried; and Case 3: those consuming baked, boiled, mashed and mixtures, fried, chips and compared them to their respective non-consumers to differentiate specific attributes of different potato forms. Interestingly, irrespective of the potato consumer definition used, the consumers always had better diet quality, nutrient intake, and adequacy.

Potato consumers (by all three definitions) always had better diet quality, albeit in the 1.6–4.7% range) as assessed using HEI 2015. HEI is a validated measure of diet quality and is indicative of compliance/adherence of a person’s diet to the eating pattern recommended by the Dietary Guidelines for Americans [29,44] and is commonly used to evaluate diets and dietary interventions [45,46,47], food environments [45], to assess changes in the diet quality over time [46,47], and to validate other nutrition research tools and indexes [48]. It has also been used to understand relationships between nutrients/foods/dietary patterns and health-related outcomes in scientific studies [49,50,51,52]. With this metric, a higher score is indicative of compliance/adherence to dietary recommendations using 13 components (nine for adequacy and four for moderation), each of which relates to key recommendations of the 2015–2020 Dietary guidelines for Americans [29]. In the present analysis, potato consumers of baked, boiled, mashed and mixtures (Case 1) compared to non-consumers had a 2.1-point higher HEI 2015 total score and the score gradually increased with increasing intake quartiles. HEI 2015 total scores were also higher for potato consumers of baked, boiled, mashed and mixtures, fried (Case 2) and for consumers of baked, boiled, mashed and mixtures, fried, chips (Case 3) than their respective non-consumers; however, the difference was smaller (+0.9 and +0.7, respectively). Additionally, compared to Case 1 consumers, Case 2 and Case 3 consumers had more HEI 2015 subcomponent scores lower than non-consumers (2 subcomponents for Case 1 vs. 6 subcomponents for Case 2 and 7 subcomponents for Case 3) suggesting that diet quality for consumers was influences by other foods commonly consumed with fried potatoes and potato chips. However, in all cases, the total HEI score remained below 50 points (out of a possible 100) and indicates the need for broad improvement on many dietary quality components.

Potato consumers had significantly higher intakes of dietary fiber, copper, magnesium, phosphorus, potassium, selenium, zinc, niacin, vitamin B6, vitamin C, vitamin E, vitamin K and total choline, and their intake increased with increasing potato intake quartiles. Additionally, potato consumers also had higher nutrient adequacy for copper, magnesium, phosphorus, potassium, zinc, thiamine, niacin, vitamin B_6_, vitamin B_12_, vitamin C, and vitamin K than non-consumers. Many of these nutrients are currently under-consumed, and especially dietary fiber and potassium are identified as “Dietary Components of Public Health Concern for Underconsumption” due to their inadequate intake [44]. Additionally, Dietary Guidelines for Americans, 2020–2025 [44] indicated that low intake of nutrient dense foods within food groups has led to low intakes of phosphorus, magnesium, and choline. Higher intakes of micronutrients among potato consumers, as observed in the present analysis, were also reported in earlier cross-sectional studies from both US and international cohorts [19,20,26,53]. Since potatoes are a good source of several of the above nutrients [2,3,4], potato consumption is naturally expected to lead to more nutrient dense diets and greater adequacy for nutrients.

Although potato consumers had higher dietary fiber intake than non-consumers, the difference was about 1 g or less and % above AI remained very low (less than 1% population above AI). On the other hand, potato consumption was associated with a 300–400 mg/d increase in potassium and the % of the population above the AI increased 17–25 percentage points. Similarly, potato consumption was also associated with significant and substantial increase in intake and population adequacy for copper, magnesium, phosphorus, vitamin B_6_, vitamin C, and vitamin K. However, potato consumers also had 130–230 more calories (kcal) than non-consumers. Substantially higher intake and higher adequacy of these nutrients among potato consumers indicates that regular inclusion of potatoes in the diet might be an effective strategy to improve the nutritional status of these nutrients.

However, potato consumption was also associated with increased sodium intake. While sodium is an essential nutrient, average intakes of sodium is high across US population compared to the Chronic Disease Risk Reduction Levels [44]. However, starchy vegetables, including potatoes, are a relatively minor source of sodium contributing to about 4% intake, compared to sandwiches or grain-based mixed dishes contributing to 21% and 8% intakes, respectively [44].

The strengths of this study include the use of a large nationally representative sample of adolescents achieved by combining several sets of NHANES data releases and the use of numerous covariates to adjust data to remove potential confounding; however, even with these covariates, some residual confounding may still exist. A major limitation of this study is the use of a cross-sectional study design, which cannot be used to determine cause and effect. The self-reported dietary recalls for dietary intake data relying on memory are potentially subject to reporting bias.

## 5. Conclusions

The results show that potato consumption was associated with better diet quality, higher nutrient intake, and improved nutrient adequacy among adolescents. Potatoes are nutrient rich foods and are a major contributor of starchy vegetables. Current intake of vegetables, including starchy vegetables, are less than the recommended levels [39]. Encouraging potato consumption, preferably without a lot of extra fat/sodium, may be an effective strategy for improving intakes and adequacy of vegetables and certain nutrients and achieving a healthier dietary pattern.

## Figures and Tables

**Table 1:** Demographics associated with potato consumption in adolescents (9–18 years of age, *n* = 16,633), NHANES 2001–2018.

	Case 1 (Baked, Boiled, and Mashed and Mixtures)			Case 2 (Baked, Boiled, Mashed and Mixtures, and Fried Potatoes)			Case 3 (Baked, Boiled, Mashed and Mixtures, Fried Potatoes, and Potato Chips)		
	Non-Consumer	Consumer	*p* Value	Non-Consumer	Consumer	*p* Value	Non-Consumer	Consumer	*p* Value
Sample N	14,102	2531		9455	7178		7228	9405	
Mean Age (years)	13.6 ± 0.04	13.4 ± 0.1	0.1289	13.5 ± 0.04	13.6 ± 0.1	0.5562	13.6 ± 0.1	13.5 ± 0.1	0.5546
Gender (% Male)	51.3 ± 0.7	46.0 ± 1.4	0.0006	52.2 ± 0.9	48.1 ± 1.0	0.0031	52.2 ± 0.9	49.0 ± 0.9	0.0192
Ethnicity									
Mexican American (%)	14.2 ± 0.9	11.6 ± 1.2	0.0037	14.9 ± 1.1	12.3 ± 0.8	0.0021	15.5 ± 1.1	12.4 ± 0.9	0.0003
Other Hispanic (%)	6.69 ± 0.58	4.79 ± 0.64	0.0036	6.90 ± 0.64	5.64 ± 0.56	0.0306	7.17 ± 0.68	5.73 ± 0.52	0.0106
non-Hispanic White (%)	56.0 ± 1.4	65.0 ± 2.0	<0.0001	56.6 ± 1.5	58.9 ± 1.6	0.0760	56.7 ± 1.6	58.2 ± 1.6	0.2819
non-Hispanic Black (%)	14.6 ± 0.8	12.2 ± 1.1	0.0060	13.0 ± 0.8	15.8 ± 1.0	0.0001	11.8 ± 0.8	16.1 ± 1.0	<0.0001
Other (%)	8.45 ± 0.51	6.50 ± 0.77	0.0130	8.67 ± 0.56	7.37 ± 0.56	0.0296	8.78 ± 0.63	7.59 ± 0.5	0.0459
Poverty Income Ratio									
<1.35 (%)	32.2 ± 1.1	30.4 ± 1.8	0.3077	32.1 ± 1.2	31.6 ± 1.2	0.6925	31.9 ± 1.3	31.9 ± 1.2	0.9704
1.35-1.85 (%)	10.8 ± 0.5	11.3 ± 1.0	0.6185	10.8 ± 0.6	10.9 ± 0.6	0.9480	10.6 ± 0.7	11.1 ± 0.6	0.5288
>1.85 (%)	57.0 ± 1.2	58.3 ± 1.7	0.4642	57.0 ± 1.3	57.5 ± 1.3	0.7167	57.5 ± 1.4	57.1 ± 1.3	0.7703
Education									
<High School (%)	98.6 ± 0.2	98.1 ± 0.5	0.3469	98.8 ± 0.2	98.1 ± 0.3	0.0204	98.9 ± 0.2	98.2 ± 0.2	0.0277
High School < Bachelor Degree (%)	1.42 ± 0.16	1.91 ± 0.49	0.3469	1.19 ± 0.16	1.92 ± 0.27	0.0204	1.15 ± 0.17	1.78 ± 0.23	0.0277
Physical Activity									
Sedentary (%)	14.5 ± 0.5	14.2 ± 1.0	0.8119	14.4 ± 0.6	14.4 ± 0.6	0.9965	14.7 ± 0.7	14.2 ± 0.6	0.5265
Moderate (%)	24.6 ± 0.6	24.9 ± 1.3	0.8601	24.6 ± 0.7	24.7 ± 0.8	0.9820	24.4 ± 0.9	24.8 ± 0.8	0.7157
Vigorous (%)	60.9 ± 0.7	60.9 ± 1.5	0.9982	60.9 ± 0.8	60.9 ± 0.9	0.9809	60.9 ± 1.0	61.0 ± 0.9	0.9163
Smoking Never (%)	79.1 ± 0.6	82.3 ± 1.7	0.0702	79.9 ± 0.8	79.4 ± 0.9	0.6578	79.3 ± 0.8	79.9 ± 0.8	0.5726
Smoking Current (%)	3.78 ± 0.35	3.01 ± 0.60	0.2444	3.88 ± 0.42	3.33 ± 0.37	0.2695	4.03 ± 0.51	3.34 ± 0.34	0.2338
Overweight (%)	16.9 ± 0.4	14.7 ± 1.0	0.0439	16.7 ± 0.5	16.3 ± 0.7	0.6819	16.9 ± 0.6	16.2 ± 0.6	0.4541
Obese (%)	19.3 ± 0.7	18.3 ± 1.0	0.3711	18.4 ± 0.7	20.2 ± 0.9	0.0788	18.4 ± 0.7	19.8 ± 0.8	0.1729

Data is presented as Mean ± Standard Error. “Other Hispanic” and “Other” are not sampled in a way to be nationally representative.

**Table 2:** Healthy Eating Index (HEI) 2015 and sub-component scores in adolescents (9–18 years of age, *n* = 16,633) potato (Case 1; baked, boiled, mashed and mixtures) consumers and non-consumers, and by intake quartiles, NHANES 2001–2018; gender combined data.

HEI 2015 Components	Non-Consumers	Consumers	*p* Value	Potato Intake Quartiles for Consumers (Cup Equivalents)				
				Q1 (<0.50)	Q2 (0.50 to <0.59)	Q3 (0.59 to <0.69)	Q4 (≥0.69)	P_quartile trend_
Total score	44.7 ± 0.2	46.8 ± 0.4	<0.0001	46.8 ± 0.7	45.3 ± 0.7	46.5 ± 0.7	48.4 ± 0.8	<0.0001
Component 1 (total vegetables)	2.06 ± 0.02	3.08 ± 0.04	<0.0001	2.60 ± 0.09	2.94 ± 0.07	3.26 ± 0.09	3.52 ± 0.11	<0.0001
Component 2 (greens and beans)	0.88 ± 0.03	0.85 ± 0.05	0.6393	1.02 ± 0.10	0.75 ± 0.11	0.83 ± 0.11	0.78 ± 0.10	0.3384
Component 3 (total fruit)	2.07 ± 0.04	2.11 ± 0.06	0.5131	2.05 ± 0.13	1.89 ± 0.13	2.09 ± 0.12	2.39 ± 0.13	0.1010
Component 4 (whole fruit)	1.82 ± 0.04	1.85 ± 0.06	0.6642	1.86 ± 0.14	1.48 ± 0.14	1.81 ± 0.13	2.26 ± 0.15	0.1095
Component 5 (whole grains)	2.09 ± 0.04	2.12 ± 0.10	0.7451	2.16 ± 0.20	1.95 ± 0.18	2.04 ± 0.19	2.33 ± 0.20	0.5762
Component 6 (dairy)	6.42 ± 0.05	6.06 ± 0.10	0.0001	6.08 ± 0.18	6.57 ± 0.19	5.82 ± 0.21	5.76 ± 0.22	<0.0001
Component 7 (total protein foods)	3.58 ± 0.03	3.91 ± 0.05	<0.0001	4.04 ± 0.08	3.93 ± 0.07	3.81 ± 0.10	3.85 ± 0.11	<0.0001
Component 8 (seafood and plant protein)	1.53 ± 0.03	1.51 ± 0.07	0.8394	1.57 ± 0.11	1.42 ± 0.11	1.44 ± 0.12	1.62 ± 0.17	0.9606
Component 9 (fatty acid ratio)	4.09 ± 0.05	4.05 ± 0.13	0.7803	4.37 ± 0.23	3.85 ± 0.22	4.00 ± 0.21	4.00 ± 0.25	0.5025
Component 10 (sodium)	4.75 ± 0.05	4.27 ± 0.10	<0.0001	4.45 ± 0.19	4.18 ± 0.21	4.41 ± 0.19	4.05 ± 0.19	<0.0001
Component 11 (refined grain)	4.72 ± 0.05	5.86 ± 0.11	<0.0001	5.55 ± 0.23	5.45 ± 0.23	6.27 ± 0.19	6.16 ± 0.25	<0.0001
Component 12 (saturated fat)	5.54 ± 0.05	5.60 ± 0.11	0.6196	5.75 ± 0.23	5.46 ± 0.21	5.54 ± 0.18	5.63 ± 0.24	0.7499
Component 13 (added sugar)	5.21 ± 0.05	5.51 ± 0.12	0.0177	5.30 ± 0.19	5.48 ± 0.21	5.19 ± 0.22	6.04 ± 0.19	0.0031

Data adjusted for age, gender, and ethnicity; and presented as Least Square Mean ± Standard Error.

**Table 3:** Healthy Eating Index (HEI) 2015 and sub-component scores associated with potato consumption in adolescents (9–18 years of age, *n* = 16,633)-NHANES 2001–2018; gender combined data.

HEI 2015 Components	Case 2 (Baked, Boiled, Mashed and Mixtures, and Fried Potatoes)			Case 3 (Baked, Boiled, Mashed and Mixtures, Fried Potatoes and Potato Chips)		
	Non-Consumers	Consumers	*p* Value	Non-Consumers	Consumers	*p* Value
Total score	44.7 ± 0.2	45.6 ± 0.2	0.0062	44.7 ± 0.3	45.4 ± 0.2	0.0346
Component 1 (total vegetables)	1.93 ± 0.03	2.64 ± 0.03	<0.0001	1.81 ± 0.03	2.57 ± 0.02	<0.0001
Component 2 (greens and beans)	0.95 ± 0.03	0.76 ± 0.04	<0.0001	1.02 ± 0.04	0.75 ± 0.03	<0.0001
Component 3 (total fruit)	2.18 ± 0.04	1.93 ± 0.04	<0.0001	2.18 ± 0.05	1.99 ± 0.04	0.0012
Component 4 (whole fruit)	1.94 ± 0.05	1.68 ± 0.05	<0.0001	1.96 ± 0.05	1.73 ± 0.05	0.0002
Component 5 (whole grains)	2.27 ± 0.05	1.85 ± 0.06	<0.0001	2.39 ± 0.06	1.86 ± 0.05	<0.0001
Component 6 (dairy)	6.60 ± 0.06	6.03 ± 0.07	<0.0001	6.69 ± 0.07	6.09 ± 0.06	<0.0001
Component 7 (total protein foods)	3.52 ± 0.04	3.78 ± 0.03	<0.0001	3.53 ± 0.04	3.72 ± 0.03	0.0002
Component 8 (seafood and plant protein)	1.60 ± 0.03	1.41 ± 0.05	0.0004	1.62 ± 0.04	1.44 ± 0.04	0.0007
Component 9 (fatty acid ratio)	3.81 ± 0.05	4.44 ± 0.08	<0.0001	3.70 ± 0.06	4.38 ± 0.07	<0.0001
Component 10 (sodium)	4.64 ± 0.06	4.70 ± 0.07	0.4611	4.59 ± 0.07	4.73 ± 0.06	0.0882
Component 11 (refined grain)	4.40 ± 0.06	5.61 ± 0.07	<0.0001	4.27 ± 0.07	5.42 ± 0.06	<0.0001
Component 12 (saturated fat)	5.57 ± 0.05	5.53 ± 0.07	0.5763	5.55 ± 0.06	5.55 ± 0.06	0.9781
Component 13 (added sugar)	5.33 ± 0.07	5.18 ± 0.07	0.1064	5.41 ± 0.07	5.15 ± 0.07	0.0073

Data adjusted for age, gender and ethnicity; and presented as Least Square Mean ± Standard Error.

**Table 4:** Covariate (age, gender, and ethnicity) adjusted energy and nutrient intakes in adolescents (9–18 years of age, *n* = 16,633) potato (Case 1; baked, boiled, mashed and mixtures) consumers and non-consumers, by intake quartiles, NHANES 2001–2018; gender combined data.

	Non-Consumers	Consumers	*p* Value	Potato Intake Quartiles for Consumers (Cup Equivalents)				
				Q1 (<0.50)	Q2 (0.50 to <0.59)	Q3 (0.59 to <0.69)	Q4 (≥0.69)	P_quartile trend_
Energy (kcal)	2094 ± 12	2225 ± 24	<0.0001	2187 ± 41	2189 ± 49	2157 ± 48	2366 ± 61	<0.0001
Carbohydrate (gm)	276 ± 2	291 ± 3	0.0002	286 ± 6	286 ± 7	283 ± 7	308 ± 8	0.0001
Dietary fiber (gm)	14.1 ± 0.1	15.3 ± 0.2	<0.0001	14.6 ± 0.4	14.5 ± 0.4	14.5 ± 0.4	17.5 ± 0.5	<0.0001
Protein (gm)	73.9 ± 0.5	82.8 ± 1.3	<0.0001	80.4 ± 1.8	82.1 ± 2.2	77.9 ± 2.0	90.7 ± 3.0	<0.0001
Calcium (mg)	1029 ± 10	1023 ± 18	0.7320	1001 ± 31	1052 ± 33	960 ± 34	1080 ± 40	0.9113
Copper (mg)	1.05 ± 0.01	1.18 ± 0.02	<0.0001	1.10 ± 0.03	1.12 ± 0.04	1.15 ± 0.03	1.35 ± 0.05	<0.0001
Iron (mg)	15.2 ± 0.1	15.5 ± 0.2	0.2581	15.3 ± 0.4	15.5 ± 0.4	14.8 ± 0.5	16.5 ± 0.5	0.1380
Magnesium (mg)	242 ± 2	267 ± 4	<0.0001	253 ± 6	259 ± 6	254 ± 6	302 ± 10	<0.0001
Phosphorus (mg)	1315 ± 9	1402 ± 19	<0.0001	1372 ± 30	1405 ± 34	1327 ± 36	1502 ± 44	<0.0001
Potassium (mg)	2205 ± 17	2617 ± 34	<0.0001	2382 ± 50	2471 ± 58	2521 ± 57	3086 ± 82	<0.0001
Selenium (µg)	102 ± 1	109 ± 2	0.0003	108 ± 3	109 ± 3	101 ± 3	119 ± 4	0.0004
Sodium (mg)	3363 ± 26	3665 ± 48	<0.0001	3578 ± 80	3625 ± 103	3493 ± 72	3959 ± 116	<0.0001
Zinc (mg)	11.0 ± 0.1	12.0 ± 0.2	<0.0001	11.3 ± 0.3	11.8 ± 0.3	11.6 ± 0.4	13.3 ± 0.5	<0.0001
Vitamin A (RE) (μg)	584 ± 7	640 ± 17	0.0013	610 ± 22	632 ± 33	605 ± 29	711 ± 47	0.0017
Vitamin B_6_ (mg)	1.78 ± 0.02	2.15 ± 0.03	<0.0001	1.96 ± 0.05	2.04 ± 0.07	2.07 ± 0.06	2.53 ± 0.09	<0.0001
Vitamin B_12_ (µg)	5.12 ± 0.07	5.30 ± 0.11	0.1633	5.13 ± 0.17	5.09 ± 0.18	4.96 ± 0.20	6.01 ± 0.33	0.0515
Thiamin (mg)	1.65 ± 0.01	1.72 ± 0.03	0.0126	1.67 ± 0.04	1.72 ± 0.05	1.63 ± 0.05	1.87 ± 0.06	0.0046
Riboflavin (mg)	2.08 ± 0.02	2.15 ± 0.03	0.0690	2.13 ± 0.05	2.15 ± 0.06	2.01 ± 0.06	2.29 ± 0.08	0.0483
Niacin (mg)	23.0 ± 0.2	25.7 ± 0.4	<0.0001	24.7 ± 0.6	25.2 ± 0.9	24.4 ± 0.7	28.6 ± 1.1	<0.0001
Folate, DFE (µg)	557 ± 6	554 ± 14	0.8296	550 ± 19	588 ± 36	527 ± 22	549 ± 24	0.6519
Vitamin C (mg)	73.9 ± 1.4	91.0 ± 3.6	<0.0001	78.6 ± 4.3	85.0 ± 5.2	90.4 ± 4.9	109.6 ± 11.6	<0.0001
Vitamin D (D2 + D3) (µg)	5.33 ± 0.08	5.52 ± 0.15	0.2182	5.33 ± 0.25	5.62 ± 0.30	5.08 ± 0.25	6.06 ± 0.36	0.1189
Vitamin E (ATE) (mg)	7.10 ± 0.10	7.45 ± 0.17	0.0834	7.50 ± 0.35	6.81 ± 0.35	7.27 ± 0.37	8.21 ± 0.44	0.0437
Vitamin K (μg)	64.9 ± 1.3	79.3 ± 3.7	0.0003	81.0 ± 7.4	61.2 ± 3.4	84.5 ± 10.8	90.2 ± 7.3	0.0002
Total choline (mg)	262 ± 3	301 ± 6	<0.0001	292 ± 10	293 ± 8	272 ± 8	343 ± 14	<0.0001

Data presented as Least Square Mean ± Standard Error. RE: retinol equivalent; DFE: dietary folate equivalent; ATE: alpha tocopherol equivalent.

**Table 5:** Energy and nutrients intake associated with potato consumption in adolescents (9–18 years of age, *n* = 16,633), NHANES 2001–2018; gender combined data.

	Case 2 (Baked, Boiled, Mashed and Mixtures, and Fried Potatoes)			Case 3 (Baked, Boiled, Mashed and Mixtures, Fried Potatoes and Potato Chips)		
	Non-Consumers	Consumers	*p* Value	Non-Consumers	Consumers	*p* Value
Energy (kcal)	2017 ± 13	2249 ± 17	<0.0001	1985 ± 15	2220 ± 15	<0.0001
Carbohydrate (gm)	268 ± 2	293 ± 2	<0.0001	264 ± 2	290 ± 2	<0.0001
Dietary fiber (gm)	13.9 ± 0.1	14.8 ± 0.2	<0.0001	13.9 ± 0.2	14.6 ± 0.1	0.0005
Protein (gm)	72.7 ± 0.6	79.2 ± 0.8	<0.0001	72.6 ± 0.7	77.7 ± 0.7	<0.0001
Calcium (mg)	1037 ± 12	1017 ± 13	0.2000	1038 ± 13	1021 ± 11	0.2560
Copper (mg)	1.03 ± 0.01	1.12 ± 0.01	<0.0001	1.02 ± 0.01	1.11 ± 0.01	<0.0001
Iron (mg)	15.3 ± 0.2	15.2 ± 0.2	0.8170	15.3 ± 0.2	15.2 ± 0.1	0.5838
Magnesium (mg)	241 ± 2	255 ± 3	<0.0001	239 ± 2	253 ± 2	<0.0001
Phosphorus (mg)	1295 ± 11	1378 ± 13	<0.0001	1291 ± 12	1361 ± 11	<0.0001
Potassium (mg)	2124 ± 18	2480 ± 25	<0.0001	2062 ± 22	2444 ± 21	<0.0001
Selenium (μg)	102 ± 1	105 ± 1	0.0206	102 ± 1	105 ± 1	0.0504
Sodium (mg)	3267 ± 26	3614 ± 35	<0.0001	3233 ± 32	3559 ± 31	<0.0001
Zinc (mg)	10.9 ± 0.1	11.6 ± 0.1	<0.0001	10.9 ± 0.1	11.5 ± 0.1	0.0002
Vitamin A (RE) (μg)	605.5 ± 8.7	577.9 ± 9.6	0.0174	613 ± 10	578 ± 9	0.0028
Thiamin (mg)	1.66 ± 0.02	1.67 ± 0.02	0.7206	1.65 ± 0.02	1.67 ± 0.02	0.3245
Riboflavin (mg)	2.09 ± 0.02	2.10 ± 0.02	0.7829	2.10 ± 0.03	2.09 ± 0.02	0.9084
Niacin (mg)	22.4 ± 0.2	24.9 ± 0.3	<0.0001	22.3 ± 0.3	24.5 ± 0.2	<0.0001
Folate, DFE (μg)	570 ± 7	539 ± 8	0.0026	572 ± 8	544 ± 7	0.0044
Vitamin B_6_ (mg)	1.73 ± 0.02	1.99 ± 0.02	<0.0001	1.71 ± 0.03	1.95 ± 0.02	<0.0001
Vitamin B_12_ (μg)	5.10 ± 0.08	5.21 ± 0.07	0.2476	5.12 ± 0.09	5.17 ± 0.07	0.6395
Vitamin C (mg)	74.1 ± 1.6	80.7 ± 1.9	0.0033	72.1 ± 1.6	80.7 ± 1.7	0.0001
Vitamin D (D2 + D3) (μg)	5.45 ± 0.10	5.26 ± 0.10	0.1158	5.47 ± 1.0	5.29 ± 0.09	0.1232
Vitamin E (ATE) (mg)	6.99 ± 0.13	7.39 ± 0.09	0.0116	6.60 ± 0.16	7.60 ± 0.10	<0.0001
Vitamin K (μg)	64.8 ± 1.7	70.8 ± 1.9	0.0103	65.9 ± 2.0	68.6 ± 1.6	0.2785
Total choline (mg)	256.2 ± 3.0	284.7 ± 3.6	<0.0001	258 ± 4	277 ± 3	0.0001

Data adjusted for age, gender and ethnicity; and presented as Least Square Mean ± Standard Error. RE: retinol equivalent; DFE: dietary folate equivalent; ATE: alpha tocopherol equivalent.

**Table 6:** Nutrient inadequacy/adequacy in adolescent (9–18 years of age, *n* = 16,633) potato consumers and non-consumers (NHANES 2001–2018, gender combined data).

	Case 1 (Baked, Boiled, and Mashed and Mixtures)			Case 2 (Baked, Boiled, Mashed and Mixtures, and Fried Potatoes)			Case 3 (Baked, Boiled, Mashed and Mixtures, Fried Potatoes, and Potato Chips)		
	Non-Consumers	Consumers	*p* Value	Non-Consumers	Consumers	*p* Value	Non-Consumers	Consumers	*p* Value
	% Population below Estimated Average Requirement (EAR)								
Carbohydrate	0.13 ± 0.04	0.01 ± 0.01	0.0026	0.18 ± 0.06	0.03 ± 0.01	0.0154	0.22 ± 0.08	0.03 ± 0.01	0.0206
Protein	1.43 ± 0.30	0.19 ± 0.15	0.0002	1.85 ± 0.45	0.41 ± 0.14	0.0025	1.98 ± 0.56	0.62 ± 0.16	0.0203
Calcium	61.9 ± 1.0	63.4 ± 2.1	0.5212	60.5 ± 1.2	64.4 ± 1.40	0.0313	60.1 ± 1.3	63.7 ± 1.2	0.0430
Copper	7.16 ± 0.65	1.46 ± 0.42	<0.0001	7.93 ± 0.85	3.57 ± 0.45	<0.0001	9.16 ± 1.04	3.90 ± 0.38	<0.0001
Iron	4.01 ± 0.37	2.65 ± 0.36	0.0084	4.15 ± 0.48	3.14 ± 0.29	0.0726	4.53 ± 0.57	3.16 ± 0.28	0.0306
Magnesium	59.5 ± 0.8	48.1 ± 1.7	<0.0001	59.3 ± 0.9	55.5 ± 1.07	0.0071	59.9 ± 1.1	56.0 ± 0.9	0.0054
Phosphorus	27.4 ± 1.0	18.8 ± 2.5	0.0011	29.3 ± 1.2	21.4 ± 1.3	<0.0001	29.3 ± 1.4	23.1 ± 1.19	0.0006
Selenium	0.40 ± 0.11	0.04 ± 0.06	0.0042	0.43 ± 0.15	0.16 ± 0.07	0.1003	0.54 ± 0.20	0.17 ± 0.06	0.0774
Zinc	15.1 ± 1.1	8.8 ± 1.6	0.0015	16.8 ± 1.3	10.4 ± 1.1	0.0002	17.2 ± 1.5	11.5 ± 1.1	0.0023
Vitamin A	41.5 ± 1.1	30.0 ± 2.9	0.0002	37.9 ± 1.3	42.0 ± 1.5	0.0461	37.3 ± 1.5	41.6 ± 1.3	0.0294
Thiamin	3.64 ± 0.51	1.10 ± 0.37	0.0001	3.90 ± 0.62	2.10 ± 0.38	0.0137	4.83 ± 0.71	1.99 ± 0.37	0.0004
Riboflavin	2.47 ± 0.41	0.87 ± 0.30	0.0017	2.82 ± 0.50	1.41 ± 0.32	0.0166	2.81 ± 0.61	1.66 ± 0.28	0.0850
Niacin	1.08 ± 0.27	0.11 ± 0.08	0.0005	1.68 ± 0.43	0.15 ± 0.07	0.0005	2.08 ± 0.53	0.24 ± 0.08	0.0006
Folate, DFE	7.42 ± 0.84	5.75 ± 1.12	0.2337	6.96 ± 0.88	7.15 ± 0.82	0.8771	7.56 ± 1.00	6.82 ± 0.78	0.5621
Vitamin B_6_	6.12 ± 0.79	0.30 ± 0.16	<0.0001	7.85 ± 1.09	1.37 ± 0.35	<0.0001	8.80 ± 1.29	1.94 ± 0.39	<0.0001
Vitamin B_12_	2.58 ± 0.45	1.37 ± 0.44	0.0531	3.11 ± 0.57	1.48 ± 0.39	0.0174	3.33 ± 0.69	1.72 ± 0.35	0.0387
Vitamin C	32.1 ± 1.4	17.2 ± 2.2	<0.0001	31.6 ± 1.7	26.3 ± 1.6	0.0224	33.3 ± 2.0	26.3 ± 1.5	0.0058
Vitamin D	91.9 ± 0.6	91.3 ± 1.2	0.6822	90.6 ± 0.8	93.1 ± 0.8	0.0270	90.3 ± 0.9	92.9 ± 0.7	0.0226
Vitamin E	88.3 ± 1.1	88.5 ± 2.1	0.9446	87.6 ± 1.3	89.5 ± 1.3	0.3225	90.2 ± 1.5	87.3 ± 1.3	0.1381
	% Population above Adequate Intake (AI)								
Dietary fiber	0.44 ± 0.10	0.31 ± 0.13	0.4215	0.57 ± 0.13	0.26 ± 0.08	0.0469	0.65 ± 0.18	0.33 ± 0.08	0.1037
Potassium	28.3 ± 1.0	53.1 ± 2.5	<0.0001	25.1 ± 1.1	42.4 ± 1.5	<0.0001	22.7 ± 1.2	40.3 ± 1.3	<0.0001
Sodium	99.6 ± 0.1	100 ± 0.04	0.0006	99.5 ± 0.2	99.9 ± 0.03	0.0020	99.2 ± 0.2	99.9 ± 0.03	0.0013
Vitamin K	37.2 ± 1.6	54.4 ± 3.5	<0.0001	36.4 ± 1.8	46.2 ± 2.2	0.0007	37.0 ± 2.2	42.8 ± 1.9	0.0430
Total choline	5.45 ± 0.60	6.72 ± 1.43	0.4122	5.96 ± 0.76	5.25 ± 0.80	0.5189	6.35 ± 0.91	5.25 ± 0.67	0.3302

DFE: dietary folate equivalent.

## Data Availability

The datasets analyzed in this study are available in the Center for Disease Control and Prevention repository; available online: http://www.cdc.gov/nchs/nhanes/. (accessed on 11 December 2020).
